# Rural health care worker wellness during COVID-19: Compassion fatigue, compassion satisfaction & utilization of wellness resources

**DOI:** 10.1371/journal.pone.0295020

**Published:** 2023-12-08

**Authors:** Bridget C. Bailey, Stephanie Cox, Lisa Terris, Dorothy van Oppen, Janie Howsare, James H. Berry, Erin L. Winstanley

**Affiliations:** 1 Department of Behavioral Medicine and Psychiatry, School of Medicine and Rockefeller Neuroscience Institute, West Virginia University, Morgantown, WV, United States of America; 2 School of Social Work, Eberly College of Arts and Sciences, West Virginia University, Morgantown, WV, United States of America; 3 Department of Neuroscience, School of Medicine, West Virginia University, Morgantown, WV, United States of America; University of Huelva: Universidad de Huelva, SPAIN

## Abstract

**Objective:**

The goal of this study was to identify factors associated with compassion fatigue (CF) and compassion satisfaction (CS) among rural health care workers (HCWs) during the COVID-19 pandemic. The secondary purpose was to assess utilization of wellness resources and preferences for new resources.

**Methods:**

A survey was distributed (October-December 2020) and completed by faculty, clinicians and staff (n = 406) at a rural university. Measures included a modified version of the Professional Quality of Life Scale (PROQOL-21), the Patient Health Questionnaire-4 and the Brief Resilience Coping Scale. Respondents reported their use of wellness resources and their preferences for new resources.

**Results:**

The mean CF score was 21.1, the mean CS score was 26.8 and 42.0% screened positive for depression or anxiety. Few of the existing wellness resources were utilized and respondents’ preferences for new wellness resources included time off (70.7%), onsite food trucks (43.0%) and support animals (36.5%). Younger age, depression and anxiety were associated with higher CF. Older age, better mental health and resilience were associated with higher CS.

**Conclusions:**

Rural HCWs have high CF, yet few utilize wellness resources. Rural health care organizations may foster wellness by providing time off for self-care, expanding mental health services and building resilience.

## Introduction

The psychological wellness of health care workers (HCWs) is a critical component of health system performance, as it is associated with patient satisfaction, treatment outcomes, and health care costs [1]. HCW wellness has declined over the past two decades [2], with historic declines since the onset of the COVID-19 pandemic. The pandemic created unprecedented challenges for healthcare systems leading to psychological distress among HCWs. Occupational stressors included increased work demands, resource uncertainty, social isolation, changes in the delivery of health care services, and increased exposure to suffering and death [3, 4]. Since the onset of the pandemic, 18% of HCWs have quit their jobs, a much higher rate compared to other occupations [5].

Occupational wellness is a multidimensional concept that can be measured with different psychometrically validated tools. For the present study we utilized the Professional Quality of Life Scale (PROQOL) because it is intended to be used with HCWs, is widely utilized, and has strong psychometric properties [6]. The PROQOL includes subscales to measure compassion fatigue (CF), and compassion satisfaction (CS) [6]. CF, defined as emotional and physical exhaustion that results in decreased capacity for empathy and compassion for others [7–9], broadly comprises burnout (BO) and secondary traumatic stress (STS). BO is emotional exhaustion, depersonalization, and a low sense of personal accomplishment resulting from working in a stressful environment [10]. It is associated with depression, anxiety, substance use, and suicide; reduced patient safety; lower quality of care; and increased health care costs, including staff turnover [3, 11, 12]. STS, or vicarious trauma, results from learning of or witnessing the traumatic experiences of others [13]. Overall CF and specifically STS are associated with lower quality of patient care and decreased job satisfaction, workforce retention, and mental health in HCWs [9, 13, 14]. For the purpose of this study, we define the term *psychological distress* as encompassed within CF. In contrast, compassion satisfaction (CS), or the positive feeling one experiences when helping others [6], mitigates psychological distress.

The reported prevalence of psychological distress among HCWs is highly variable depending upon the health care setting, measurement tool, and timing of the assessments. Prior to the COVID-19 pandemic, studies found that 39% to 79% of HCWs had moderate-to-high CF [15–17], specifically, 38% had STS [18], and 33% to 75% reported moderate-to-high BO [12]. Recent studies suggest that the persistence of the pandemic has exacerbated psychological distress. Since the onset of the pandemic, 72% to 74% of HCWs reported experiencing psychological distress, 34% to 62% anxiety, 45% to 51% depression, and 36% insomnia [19–21]. Another study found that 41.3% of HCWs had STS and 56% experienced professional BO [22]. This is in comparison a meta-analysis of studies of psychological distress in the general population during COVID-19, which found 37.5% experienced psychological distress in general, 38.19% experienced anxiety and 34.3% depression [23].

A small body of empirical research has identified individual and professional factors associated with psychological distress and CS among HCWs during the COVID-19 pandemic [22–26]. Individual characteristics associated with higher CF included being female [7, 27, 28], single [29], and younger [24]; living with young children or elderly parents [27]; having worse physical health [22, 27]; having higher levels of depression, anxiety, or perceived stress [22, 26, 28]; and having a trauma history [27]. Professional characteristics associated with higher CF included being early career [27], being a resident versus a physician [29], being a physician versus a nurse [25], providing direct patient care [22, 27], and being exposed to patient death [22]. Protective factors, or those associated with higher CS, included being involved in direct patient care [28] and being a nurse versus a physician [25, 28].

The majority of research on psychological distress among HCWs during the pandemic has been conducted in urban areas or outside of the United States. Rural areas in the United States, however, often have less access to health care and lower COVID-19 vaccination rates, which has led to acute-care hospitals in these areas becoming overwhelmed. In rural communities designated as health care shortage areas, HCWs typically have higher patient caseloads and fewer opportunities to take time off from work. Additionally, rural patients often have more chronic, comorbid conditions and higher rates of death by suicide and drug overdose [30]. Meanwhile, rural HCWs, like the communities they serve, have limited access to mental health services and professional colleagues for support [31]. Some rural hospitals have closed due to a lack of resources, particularly during the pandemic. Thus, it is no surprise that psychological distress among rural HCWs has worsened during the pandemic due to insufficient resources [32–34], staffing shortages [34, 35], increased workloads [32, 36], and a lack of support from leadership [33, 36, 37].

Existing research on psychological distress among rural HCWs is limited and specific to a particular discipline or setting (e.g., nurses in the emergency department). Less is known about its overall impact on the rural health care workforce [38]. The primary purpose of the present study was to measure the prevalence of CF and identify factors associated with CF and CS among rural HCWs during the COVID-19 pandemic. The secondary purpose was to determine HCW utilization of existing wellness resources as well as the need to expand and enhance those resources. One review of the literature found the most common wellness and support initiatives implemented for HCWs during the COVID included expanding resources and services to address basic needs, offering training for preparedness and safety, and expanding emotional health and psychological support programs [39]. We hypothesized that the prevalence of CF was higher in rural HCWs compared to previously published studies of urban HCWs. We also hypothesized that rural HCWs with higher levels of resilience and social support would have lower CF. Identification of risk factors for psychological distress in rural HCWs will enable the development of targeted interventions that are feasible to employ in rural areas with limited resources to support HCW wellness, leading to improved health care in those areas.

## Methods

We distributed an anonymous and confidential web-based survey to faculty, staff, and/or trainees at a rural academic medical center in West Virginia in Fall 2020. Data from two departments, Psychiatry and Internal Medicine, are included in this analysis. Survey participation was optional, and respondents did not receive any financial incentives for completing the survey. We developed the survey as part of a larger initiative to assess HCW needs during the COVID-19 pandemic and determine whether existing wellness resources needed to be expanded.

### Study recruitment & consent

Chairs or designees of the departments of psychiatry and internal medicine at one rural academic medical center emailed a Web link for the survey to 764 employees. The study inclusion criteria were being 1) a current employee and 2) at least 18 years of age. There were no exclusion criteria. Employee roles included, but were not limited to clinicians (e.g., physicians, nurses, psychologists, social workers), trainees, administrative personnel, and staff. We received responses from 405 individuals (53.1% response rate). The survey was anonymous and confidential, hence written informed consent was not required. The researchers did not have access to data that would identify participants during or after data collection. The West Virginia University Institutional Review Board approved this survey (WVU Protocol #: 2009120261).

### Data collection

We developed four survey modules in the Web-based survey application REDCap. Based on pilot testing, the majority of respondents should have been able to complete the survey in less than 15 minutes. Data collection occurred from October to December 2020.

#### Module 1: Employee needs assessment core

The module assessing employee needs had 75 items, including sociodemographic characteristics (sex, age, education, relationship status, number of children in home, adult caretaking responsibilities) and occupational information (e.g., role, setting of clinical services, years of work experience). We took questions relating to marital status (i.e., What is your marital status?), educational degree (i.e., What is the highest degree or level of school you have completed?) and number of children in the household (i.e., How many children or adolescents live in your household?) from the U.S. Census Bureau’s 2020 Pulse survey. We took one question related to work productivity from the WHO Health and Work Performance Questionnaire [40]. This question states: “On a scale from 0 to 100, where 0 is the worst job performance anyone could have at your job and 100 is the performance of a top workers. How would you rate your usual job performance?” Five questions regarding quality of life, overall physical health, overall mental health, sleep quality, and fatigue were taken from the National Institute of Health’s (NIH’s) Patient-Reported Outcomes Measurement Information System (PROMIS) [41, 42]. Overall life satisfaction was measured using the Cantril ladder [43]. This question states: “Please imagine a ladder with steps numbered from 0–100. The top of the ladder (100) is the BEST possible life for you and the bottom of the ladder (0) the WORST possible life for you. On which step of the ladder would you say you stand at this time?” Screening for depression and anxiety was done using the Patient Health Questionnaire-4 (PHQ-4) [44]. This module also included 18 items from the PROQOL-21 [45], which includes the CF and CS subscales. In addition, three items asked whether the respondent had experienced the death of a family member, close friend, or a patient within the past year. Other items asked whether the respondent utilized specific existing medical center wellness resources (e.g., Faculty Staff Assistance Program, Coping with COVID: Support for Employees & Providers Facebook page); if so, how frequently since the onset of the COVID-19 pandemic; and whether they had interest (i.e., Which WVU Resources would you use?) in additional wellness resources. Finally, two open-ended questions asked what the respondent’s employer could do to support their wellness and promote a healthy work environment.

#### Module 2: COVID-19 needs assessment module

The second module included 46 items to determine how faculty, staff, and/or trainees were impacted by COVID-19, such as changes to sleep, concentration, worry, work expectations, workload, stress, and finances. Of these items, four were from the Chestnut Global Partner’s Workplace Outcome Suite [46], which the university’s internal employee assistance program (EAP) and Faculty and Staff Assistance Program (FSAP) used at the time. EAPs are an employer funded benefit that include access to confidential short-term assessment, counseling, and referral for a broad range of difficulties including problems related to work or the employees’ personal life. Most universities have an internal employee assistance program which are commonly called faculty staff assistance programs or FSAPs, which are available for all university employees and their dependents. We modified the items to reflect how the pandemic specifically had impacted the respondent’s work productivity in the past month. The module also included questions about the availability of personal protective equipment (PPE) and whether the respondent’s department had communicated with employees effectively. We took one item regarding healthy and unhealthy coping strategies from the Centers for Disease Control and Prevention (CDC). The module closed with a question regarding the respondent’s use of wellness resources outside of the organization.

#### Module 3: Resilience

To measure resilience, we used the four-item Brief Resilience Coping Scale (BRCS) [47]. A BRCS total score is generated by summing the items (range: 4–20), and respondents can be categorized as low-resilient (4–13), medium-resilient (17–20), and high-resilient copers (17–20) [47].

#### Module 4: Social support

The module on social support included the PROMIS Short Form v2.0 –Emotional Support 4a and one item from the PROMIS Short Form v2.0 –Social Isolation (“I feel isolated from others”) [42]. The Emotional Support items, per the PROMIS scoring manual, were summed to generate a total score, and the PROMIS Emotional Support conversion table was used to convert raw total scores into standardized scores (mean = 50, standard deviation = 10). The standardized scores were recoded into low (<one standard deviation below the mean), normal (within one standard deviation of the mean) and high (> one standard deviation above the mean) levels of emotional support.

### Data analysis

Stata MP 15.0 [48] was used to calculate descriptive statistics and for the multivariable models. Descriptive statistics were used to summarize the sample characteristics, impact of COVID-19, use of wellness resources, and interest in new wellness resources. Primary outcomes were mean CF and CS subscale scores on the PROQOL-21 from Module 1. We modified the PROQOL-21 scoring algorithm as three items from that instrument were not included in the present survey. In the descriptive analysis, we recorded the PROQOL-21 subscale scores as categorical variables, with *normal* defined as within one standard deviation of the mean subscale score and *low* and *high* defined as more than one standard deviation below or above the mean subscale score, respectively (a similar scoring approach is used in the original PROQOL-21). In the multivariable models, the modified PROQOL-21 total subscale scores were used. Bivariable regression models, using robust variance estimation of, were used to determine whether the following known covariates were associated with CF or CS scores in the present sample: occupational role (clinician, trainee, administrator/other), sex, early career (defined as less than five years’ experience), caretaking in their personal capacity (caring for children and/or parents), marital status (single versus not single), quality of life (fair/poor versus good/very good/excellent), overall physical health (fair/poor versus good/very good/excellent), overall mental health (fair/poor versus good/very good/excellent), symptoms of depression or anxiety (measured as a positive score on the PHQ-4), and exposure to trauma over the past year (none, either family/friend or patient/client death, or family/friend death and patient/client death). We also included variables that, to our knowledge, have not previously been investigated in studies of CF and CS: resilience (low, medium, high) and social support (low, normal, high). Given the dearth of research on CF/CS in rural HCWs, statistically significant covariates in the bivariable models were then included in the multivariable analyses. Qualitative responses (i.e., two open-ended items regarding methods for supporting HCW wellness from Module 1) were analyzed using thematic analysis. This included two of the authors reading through the responses. Responses for these two questions were combined. First, two authors independently read through the responses and generated codes capturing themes to categorize the responses. Individual comments could receive up to five codes. The authors then met to review and define themes. The codes reflected concrete requests (e.g., reduced workload, more time off from work), factors known to be associated with employee satisfaction (e.g., autonomy), and aspects of workplace culture and/or values (e.g., prioritizing employee wellness, clear communication). Next, a third author adjudicated any differences between the two authors’ codes. The codes were transformed from wide to long format, and frequencies are reported.

## Results

### Sociodemographic and professional characteristics

Of the 405 respondents, the majority were female (74.9%), had a graduate degree (71.1%), were a clinician or physician (71.4%), and were currently married (62.1%). The vast majority (84.4%) worked in direct patient care. A small number (*n* = 37 [9.1%]) were trainees, and 19.5% (*n* = 79) were administrators or did not provide direct patient care (see Table 1).

**Table 1:** Sample characteristics (*N* = 405).

Characteristic	*n* (%)
*Age* (years)	
20–29	74 (18.2)
30–39	145 (36.0)
40–49	85 (20.9)
50–59	56 (13.8)
≥ 60	45 (11.1)
*Number of years worked in current profession*	
Trainee	27 (6.7)
<1	32 (7.9)
1–2	48 (11.9)
3–5	96 (23.7)
6–10	67 (16.5)
> 10	135 (33.3)
*Occupational role*	
Trainee	37 (9.1)
Clinician	289 (71.4)
Administrator/other nonclinical role	79 (19.5)
*Overall quality of life*	
Poor/fair	44 (11.0)
Good/very good/excellent	358 (89.1)
*Overall physical health*
Poor/fair	69 (17.3)
Good/very good/excellent	329 (82.7)
*Overall mental health*	
Poor/fair	101 (25.2)
Good/very good/excellent	300 (74.8)
*Trauma exposure in past year*	
None	97 (24.6)
Death of family/friend or patient	202 (51.3)
Death of family/friend and patient	95 (24.1)
*Positive PHQ-4 screen*	
Depression	86 (21.6)
Anxiety	154 (38.3)
*CF score*	
Low (1 SD below mean)	58 (16.8)
Medium (within 1 SD of mean)	237 (68.5)
High (1 SD above mean)	51 (14.7)
*CS score*	
Low (1 SD below mean)	50 (14.8)
Medium (within 1 SD of mean)	228 (67.5)
High (1 SD above mean)	60 (17.8)
*BRCS total score*	
Low resilience	93 (27.3)
Medium resilience	169 (49.6)
High resilience	79 (23.2)
*Social support*	
Low (< 1 SD below mean score)	28 (8.4)
Normal/high (within 1 SD or mean or higher)	304 (91.6)

### Physical and mental health, quality of life, and professional quality of life

Few respondents reported fair or poor overall physical health (17.3%), mental health (25.2%), or quality of life (11.0%; see Table 1). More than a third (38.3%) screened positive for anxiety, 21.6% screened positive for depression, and 42.0% screened positive for either anxiety or depression on the PHQ-4. In terms of trauma experiences, 37.5% had a close family member or friend die in the past year, and 62.4% had a current or former patient die within the past year. The mean CF score on the modified PROQOL-21 was 21.2 (SD = 5.7), and the mean CS score was 26.8 (SD = 6.3). Less than 20% of respondents had high CF (14.7%) or CS (17.8%).

### Impact of COVID-19 on work performance and coping

The majority of respondents (50.7%) reported sleeping less since the onset of the pandemic. Only a few reported that they had missed work (16.3%), had to leave work early (13.7%), or were late for work (6.9%). Strategies to cope with the pandemic included eating more food than usual (39.9%), eating high-fat or sugary foods (31.3%), and exercising less than usual (30.5%). One-fifth (20.9%) reported drinking alcohol to cope with the pandemic, and none reported misuse of prescription drugs or illegal drug use. About half (52.6%) of respondents were concerned about contracting COVID-19 and nearly three-quarters (72.0%) reported that their stress had increased due to COVID-19 (see Table 2).

**Table 2:** Impact of COVID-19 (*N* = 405).

	Disagree % *(n)*	Neutral % *(n)*	Agree% *(n)*
I am very worried about getting COVID-19.	24.6% (86)	22.9% (80)	52.6% (184)
I have had a hard time sleeping because of COVID-19.	62.4% (217)	17.2% (60)	20.4% (71)
I have had a hard time concentrating because of COVID-19.	57.5% (200)	15.8% (55)	26.7% (93)
I am feeling overwhelmed by COVID-19.	39.5% (137)	16.1% (56)	44.4% (154)
I am worried about how to balance work and child/elder care or homeschooling.	38.0% (93)	11.0% (27)	51.0% (125)
I can count on my co-workers to help me at work if I have problems.	11.3% (39)	17.4% (60)	71.4% (247)
I feel that my department leadership is doing everything they can to protect me from getting COVID-19 at work.	19.1% (66)	17.3% (60)	63.6% (220)
My work expectations or workload has increased because of COVID-19.	22.7% (78)	23.3% (80)	53.9% (185)
My stress has increased because of COVID-19.	15.7% (55)	12.3% (43)	72.0% (252)
I have experienced benefits from working at home.	22.2% (41)	17.8% (33)	60.0% (111)
My department leadership has effectively communicated strategies used to protect staff and patients.	16.5% (57)	15.9% (55)	67.6% (234)
My department leadership has effectively communicated about working from home & telehealth services.	22.1% (63)	15.8% (45)	62.1% (177)
I have the resources (e.g., computer, internet) that I need to work from home.	9.8% (28)	7.0% (20)	83.3% (239)

*Note*. BRCS = Brief Resilience Coping Scale; CF = compassion fatigue; CS = compassion satisfaction; PHQ-4 = Patient Health Questionnaire 4.

### Utilization of existing wellness resources and preferences for new resources

More than a third of respondents (39.6%) reported using existing wellness resources at least once in the past year (see Table 3). The most frequently used existing resources were the “Coping with COVID” Facebook page (18.5%), FSAP (10.3%), and the wellness center (10.0%). The wellness center is run by a separate multidisciplinary health sciences department who provide services such as fitness and stress management and are available to all employees. The majority of respondents reported that they either “might use” or “would definitely use” the following new resources, from the most to the least popular: time off from work for self-care, onsite food trucks, time off to utilize the EAP or other wellness resources, a temporary reduction in relative value units (RVUs) (i.e., measurement for productivity of reimbursable services), increased number of EAP sessions, expansion of EAP to include psychiatric care, and a peer-support program (see Table 3).

**Table 3:** Utilization of existing wellness resources and preferences for new resources (*N* = 405).

*Use of Existing Wellness Resources*	Yes % *(n)*	No % *(n)*
“Coping with COVID” Facebook page	18.5% (74)	81.5% (326)
Faculty and Staff Assistance Program (FSAP)	10.3% (41)	89.7% (358)
Wellness center (gym)	10.0% (40)	90.0% (360)
Virtual well-being & mindfulness sessions	8.5% (34)	91.5% (367)
Employee Assistance Program (EAP)	3.7% (15)	96.3% (387)
Group stress-management sessions	0.3% (1)	99.8% (400)
Drop-in lunch group/peer support	0.5% (2)	99.5% (398)
Mental health support group	0.3% (1)	99.7% (397)
24–7 triage crisis line	0.3% (1)	99.8% (399)
Brief emotional support team session (BEST)	0.0% (0)	100.0% (401)
*Preferences for New Wellness Resources*	Might Use/Would Definitely Use	Would Not Use
Time off from work for self-care	93.9% (372)	6.1% (24)
Food trucks onsite at work	79.6% (301)	20.4% (77)
Time off to utilize EAP or other wellness resources	68.6% (267)	31.4% (122)
A temporary reduction in relative value units (RVUs)	62.9% (168)	37.1% (99)
Increased number of EAP sessions	60.6% (226)	39.4% (147)
Expansion of EAP to include psychiatric care	57.3% (216)	42.7% (161)
Buddy support system	52.9% (202)	47.1% (180)
Webinars on stress or burnout	46.4% (181)	53.6% (209)
Assistance with sick-child care	44.4% (116)	55.6% (145)
Support animals or animal therapy	32.0% (124)	68.0% (263)
Assistance with elder care or care for adults with disabilities	28.9% (79)	71.1% (194)

### Recommendations for employer actions to support HCW wellness

We summarized the open-ended responses regarding employer actions to support HCW wellness into 23 categories, or “themes,” of recommendations. Themes and example responses are provided in Table 4. The most highly endorsed themes included, “foster a connected and supportive culture, improve communication, encourage a culture of wellness, reduce workload, hire more providers/staff, and improve autonomy/schedule of work.” The theme “foster a connected and supportive culture” indicates that HCWs want to feel that they are valued/appreciated and desire a sense of community/belonging. Similarly, the theme “encourage a culture of wellness” reflects the importance of the employer’s prioritization of HCW wellness and recognition of its impact on patient care. Further, this theme indicates the need for employers to explicitly communicate that they recognize the value of work/life balance and that the responsibility for protecting against BO must shift from the individual to the organizational environment. The theme “improve autonomy/schedule of work” reflects HCWs’ desires to control their work schedules and have a transparent and accessible process to take time off or work remotely when needed as well as the need for protected time for breaks during the workday. The need for time off was frequently endorsed (*n* = 42), including time off in general (*n* = 14) or for mental health or self-care (*n* = 28).

**Table 4:** Summary of open-ended responses regarding recommendations for actions employers could take to support health care worker wellness.

Category/Theme	Example Responses
Foster connected & supportive culture (*n* = 73)	• “Raise awareness of importance of being kind and understanding of coworkers.” • “Provide more opportunity to connect with co-workers and support each other during higher stress times.” • “Encouragement and praise for a job well done.”
Improve communication (*n* = 43)	• “Direct communication with employees. Notification of changes when possible.” • “More information on what is going on.” • “Listen to our concerns.”
Encourage culture of wellness (*n* = 42)	• “Change the narrative/focus from productivity to wellness and happiness in the workplace.” • “Have mental health breaks during the day in between clinics to be able to relieve stress before continuing treatment for other patients.” • “The organization and our department can start by recognizing the true degree of burn out both pre-pandemic and currently. Implementing policies that preserve our passion, empathy, and lives are greatly needed to allow our providers to best serve and remain with our organization.”
Reduce workload (*n* = 40)	• “Reductions in productivity expectations (RVU/slot reductions).” • “Lower RVUs and increase time/opportunities for self-care during COVID.” • “I bring my lunch and eat at my desk every day just so I can keep up with e-mails or attend meetings scheduled during lunch. It is hard to take time off from work because when I return I am overwhelmed playing catch up and it is very stressful. The work load is too much.”
Hire more providers/ staff (*n* = 38)	• “Appropriate staffing based on acuity.” • “Increase in providers to mitigate recent increase in patient volume & demands.” • “More staffing to better distribute tasks.”
Improve autonomy/ schedule (*n* = 35)	• “Allow us to work from home more often.” • “Schedule flexibility.” • “Don’t make a person feel they have to sit in front of their computer a full 8.5 hours a day; that they can get up and move around (if they leave their seat it’s not a crime).”
Time off for mental health (*n* = 28)	• “Mental health days off.” • “Definitely offer ‘self-care’ days off. We used to have one personal day a year. Having two or three self-care days you can have off paid in addition to PTO we receive would be helpful. We discuss self-care and mental health as priority so much but I feel it is not always as well supported or promoted for employees.” • “Allow for mental health days.”
Improve financial remuneration (*n* = 23)	• “Increase pay/bonuses for people taking care of COVID patients.” • “Stop taking our money, provide hazard pay, and do more than provide lip service and street signs calling us ‘heroes.’ No one is buying that [stuff].” • “Fair compensation and personal days for the level of work and stress required for the job. COVID-19 has led to more work, high stress, and suspension of retirement match. Conditions continued to grow worse with no incentives to reflect employee effort.”
Do more to prevent COVID (*n* = 22)	• “Provide free COVID-19 testing for employees.” • “Enforce mask-wearing during pandemic. Better staff-to-patient ratios.” • “Without proper rules, then there is a lot of stress placed on the consulting teams to be exposed. This leads to significant unnecessary risk of exposure to residents and creates tension and stress within the department and creates animosity among departments.”
Team building (*n* = 21)	• “More supportive measures through employee engagement: more ‘fun’ distractions throughout the year in lieu of the fact that a lot of our events, meetings, luncheons have been cancelled for almost a year now.” • “Having a fun ‘virtual’ luncheon where we do fun ice breakers, or activities 1x every other month, or activities on Spirit day might be a good way to build morale and create more spirit as times have been really tough on everyone.” • “Give us time off to work with each other, team building.”
Sick leave/accessing sick leave or PTO/separate sick time (*n* = 20)	• “Split vacation time and sick leave. It is hard to make a decision to take a sick day when you would like to take a proper vacation and vice versa.” • “Offer personal days in addition to PTO. If I need to take a day off, more than likely I won’t because I don’t want to take from my vacation.” • “Dedicated PTO to use for our own personal appts, etc.”
Improved/more wellness resources (*n* = 15)	• “Bring support animals back even if we have to wear gloves to pet them.” • “Pet therapy for staff, mental health check-ins or mental health days.” • “Mindfulness training.”
More time off (*n* = 14)	• “More PTO.” • “Giving off requested days when asked and not being refused due to PTO balance and staffing.” • “More time off to balance stress and provider fatigue (many have used sick days and have little PTO). Even a day every month.”
Improve equality/equity (*n* = 14)	• “[Provide] equal treatment and more opportunity for an impaired employee to get treatment and return to their position.” • “Maintain equity and reward good behavior and work and address poor behavior or work.” • “Offer equivalent work-from-home and office-time schedules across the entire group.”
Improve physical work environment (*n* = 13)	• “Provide us with safe eating spaces.” • “Better parking situation.” • “Cleaner staff bathrooms and for patients as well.”
Supportive supervision (*n* = 10)	• “I have felt a lack of face-to-face contact with my manager, other than perfunctory meetings. With some personal experience as a manager, I feel the level of pats on the back or atta boys could be improved.” • “[I would like] for management to come in on the off shifts and seem interested in how things are.” • “[Provide] more support to staff with aggressive or verbally abusive patients.”
Less admin./paperwork (*n* = 10)	“Utilizing support staff for tasks such as pill counts, obtaining/scheduling random UDSs, educating people on no-show policy, and ensuring that individuals having MyChart video appointments have an account set up. Obtaining scribes. Refraining from filling time that could be used for charting or self-directed continued education with additional requirements.” • “Solutions for documentation and triage burden.” • “Not specific to WVU, but more support/changes to diminish typing and endless electronic record requirements/notifications that take away so much time from direct patient interaction.”
Improve childcare options (*n* = 8)	• “Access to emergency child care especially after a COVID exposure.” • “Either be flexible when children need care or have resources to help with child care.” • “Having better childcare options for employees.”
Access to mental health care for providers (*n* = 7)	• “Having supportive psychotherapy sessions for providers.” • “More mental health support for professionals.” • “Providing access to mental health professionals outside of your own work place who are mental health providers and making it transparent as to how one can access that without having to ask for it.”
More training/mentoring (*n* = 3)	• “I feel invisible and like there is no room for growth.” • “More training when given new job duties.” • “Improved access to formal mentoring program (outside of department) to help with career development to assist with personal goals & support in work environment.”
Privacy of EAP/FSAP (*n* = 3)	• “Acknowledge the tensions that are present and bring in confidential outside resources. The EAP program requires we reach out to a long-term colleague from Behavioral Medicine who is still involved in B. Med activities. No real confidentiality or trust.” “Ensuring privacy with EAP or finding outside services for [*department name*] faculty, staff and trainees.” • “Improved access for behavioral medicine providers, staff & trainees to mental health care ‘outside’ of the system. We still have contact with EAP & FSAP providers so this makes it difficult for true confidence with anonymity.”
More supplies (*n* = 3)	• “Improved support/supplies for clients. More convenient ways of getting supplies/equipment.” • “Have access to the supplies and needs of the workspace.” • “Department administrators should ensure that every employee under their umbrella has the space, equipment, supplies, needs to complete their job to the best of their ability. Obtaining things from ‘surplus’ is not always the answer.”
Reduce wellness stigma (*n* = 1)	• “Normalizing ‘mental health days’ and not feeling guilty for wanting to take time off, and motivating people actually to use their time off instead of just selling them at the end of each year.”

*Note*. EAP = Employee Assistance Program; FSAP = Faculty and Staff Assistance Program; PTO = personal time off; RVU = relative value unit; UDS = urine drug screen; WVU = West Virginia University.

### Factors associated with CF and CS

In the bivariable model, age, staff type, quality of life, physical health, mental health, PHQ-4 depression and anxiety, resilience, and emotional support were associated with CF, while only age, quality of life, physical health, mental health, PHQ-4 depression and anxiety, resilience and emotional support were associated with CS (see Table 5). In the multivariable model, a high level of emotional support and being an administrator or other support staff were associated with lower CF. Respondents aged 30−39 years old and those with a positive PHQ-4 depression or anxiety screen had higher CF. Respondents with medium or high levels of resilience and those aged 50−59 years old had higher CS in the multivariable model, while those with a positive PHQ-4 depression screen had lower CS.

**Table 5:** Bivariable and multivariable models of factors associated with Compassion Fatigue (CF) and Compassion Satisfaction (CS).

	Bivariable Models						Multivariable Models					
	CF			CS			CF			CS		
	Coef.	SE	*p*	Coef.	SE	*p*	Coef.	SE	*p*	Coef.	SE	*p*
Female	0.09	0.64	.890	0.03	0.76	.971						
Age (ref. 20–29 years)												
30–39 years	2.84	0.83	.001	−1.12	0.98	.254	3.18	0.85	.000	−0.87	1.03	.401
40–49 years	1.18	0.90	.191	0.44	1.05	.679	1.80	0.96	.061	0.20	1.04	.846
50–59 years	1.32	1.16	.258	2.57	1.16	.027	0.51	1.17	.661	3.38	1.27	.008
60+ years	−2.01	1.04	.054	2.45	1.43	.087	0.01	1.37	.993	2.59	1.51	.086
Not married	0.97	0.64	.128	0.09	0.72	.895						
Caretaker	0.50	0.62	.422	0.32	0.69	.647						
6+ years work experience	−0.05	0.31	.880	0.36	0.34	.296						
Staff type (ref. clinician)												
Administrator/other	−2.53	0.93	.007	−1.46	1.04	.160	−3.61	0.89	.000			
Trainee	−1.26	0.92	.174	1.03	1.17	.381	1.01	0.95	.286			
Trauma exposure in the past year (ref. none)												
Family/friend or patient/client death	1.40	0.76	.066	−0.08	0.94	.936	1.11	0.70	.116			
Family/friend death and patient/client death	1.8	0.93	.052	1.46	1.04	.161	0.89	0.83	.283			
Quality of life—Good/very good/excellent (ref. poor/fair)	−4.71	0.86	.000	3.87	1.06	.000	−1.88	1.01	.063	1.15	1.08	.288
Physical health—Good/very good/excellent (ref. poor/fair)	−2.65	0.76	.001	2.12	0.87	.015	1.41	0.77	.068	−1.03	0.95	.278
Mental health—Good/very good/excellent (ref. poor/fair)	−4.59	0.65	.000	4.22	0.75	.000	−1.47	0.87	.091	1.88	1.03	.067
Positive PHQ-4 depression screen	5.69	0.72	.000	−3.91	0.87	.000	2.72	0.87	.002	−2.11	1.02	.040
Positive PHQ-4 anxiety screen	5.04	0.57	.000	−3.06	0.70	.000	3.29	0.64	.000	−0.85	0.88	.335
Resilience (ref. low)												
Medium	−0.46	0.81	.572	4.34	0.85	.000	1.10	0.72	.129	3.41	0.88	.000
High	−2.64	0.97	.007	7.09	0.96	.000	−0.13	0.92	.889	6.01	1.04	.000
Emotional support (ref. low)												
Normal	−2.17	1.00	.031	0.70	1.49	.641	−1.66	1.13	.141	0.37	1.17	.755
High	−4.48	1.00	.000	3.14	1.50	.037	−2.95	1.16	.011	1.04	1.20	.385

*Note*. PHQ-4 = Patient Health Questionnaire 4.

## Discussion

Few of the rural HCWs in our sample had high CF (14.7%) or low CS (14.8%). The prevalence of CF was lower in our sample compared to those of studies conducted in China and Spain during the pandemic [19, 25] but slightly higher than that of a study conducted across the U.S. [21]. In a study conducted during the pandemic in Kansas, investigators found rates of CS similar to that in our sample, but no HCWs in that study had high CF [49]. Due to differences in measures used, it is difficult to make direct comparisons across studies. However, our data do not appear to support our hypothesis that rural HCWs have higher rates of CF. Interestingly, Sprang et al. did not find urban-rural differences in the prevalence of CF among mental health care providers in Kentucky in 2007 [50]. Despite the aforementioned challenges of delivering health care in rural areas, it is likely that the impact of the COVID-19 pandemic on HCWs is ubiquitous across settings as are staffing shortages. Further, rural HCWs may have higher CS or higher resilience that mitigates the challenges of working in resources-scarce environments.

### Factors associated with compassion fatigue and compassion satisfaction

Our results do support our hypothesis that higher levels of resilience would be associated with lower CF. In the one prior study that looked at the relationship between CF and resilience in HCWs, investigators reported that resilience mediated the relationship between CF and quality of care in nurses working in the Philippines during the COVID-19 pandemic [51]. Traditional employee wellness programs include stress-reduction programs and screening and treatment for mental health problems, but few explicitly embed strategies to build resilience. Prospective research using validated instruments to measure resilience is needed to determine whether resilience-based interventions can prevent CF in HCWs.

Our results do support our hypothesis that social support is associated with CF. In the multivariable analyses, social support was significantly associated with CF but not CS. We measured general social support, which is associated with overall physical and mental health at the individual level [3, 4, 21, 27, 31]. In our sample, 42% of respondents in our sample had high levels of emotional support, though the literature suggests that only 16% would score in this range on this standardized measure. Further evidence of high levels of social support was our finding that 71.0% of respondents reported that they could count on their coworkers to help if they had problems.

In the open-ended responses regarding things employers could do to support HCW wellness and promote a healthy workplace, many respondents expressed a desire for a sense of community at work, a supportive work environment, and other nonmonetary-based initiatives to support their well-being. Prior studies have demonstrated that supervisor and organizational support are important components of workplace wellness [52]. In other studies, perceived support from leadership was associated with reduced BO and/or fewer psychiatric symptoms in resident physicians [53–55]. As such, organizational support may also be important in reducing psychological distress in HCWs. These findings support the need to create work cultures that prioritize HCW well-being. Measures of perceived organizational support may provide a mechanism for health care organizations to evaluate whether their wellness initiatives are working and to determine whether workplace culture is improving.

Additionally, our results show younger age (30–39 years) was associated with increased CF. This is consistent with a similar study on CF in HCWs during the COVID-19 pandemic in an urban academic medical center, which found HCWs less than 40 years had increased work exhaustion, burnout, depression, anxiety and poorer well-being. Additionally, older age (50–59 years) was associated with higher CS. These findings suggest younger HCWs may have been more susceptible to CF during the COVID-19 pandemic. Possible reasons for this finding include that younger HCWs may have had more family responsibilities, such as child care, and homeschooling children out of school during the pandemic. Our results found that 51% of participants indicated they had concerns about providing childcare or eldercare during the COVID-19 pandemic. This is consistent with previous literature, which found HCWs living with children and elderly parents during the COVID 19 pandemic had higher CF [24, 27].

Our finding that higher depression and anxiety were associated with higher CF and lower CS is also consistent with the literature [22, 26]. Specifically, a cross-sectional survey of 184 HCWs from 45 different countries during the COVID-19 pandemic found higher psychological distress, depression and anxiety were associated with increased STS and BO [22]. This suggests that universally, across countries and urban and rural areas, HCWs had increased rates of anxiety and depression during COVID-19 and those with high to moderate depression and anxiety were more vulnerable to CF and less CS. HCWs during COVID-19 may have benefited from time off to utilize of occupational mental health supports. While our results show only 14% of HCWs used formal mental health supports (i.e., EAP and FSAP) during the COVID-19 pandemic, the majority indicated they would use EAP and other wellness resources if given time off to access these resources. More generally, 93.9% indicated they might or would use time off for self-care indicating that more time to use available mental health supports may potentially decrease CF and psychological distress.

### Wellness resources for rural HCWs

The highest utilized existing Wellness Resource was the “Coping with COVID” Facebook page which was quickly developed and rolled out early in the pandemic. The Facebook group is accessible to anyone affiliated with the health care system and is used to share information about wellness resources, highlight local “health care heroes,” and share stories of support and encouragement. As of January 2022, it had more than 7,200 members.

As discussed above many respondents expressed the need for more time off for self-care, sick leave, or to utilize wellness resources. Employees, particularly early in the pandemic, may have quickly depleted their sick leave due to quarantine in response to suspected or confirmed exposure to COVID-19. Those caring for young children, disabled adults, or elderly family members also likely faced increased need for time off during the pandemic. Organizations may want to consider implementing policies that would automatically expand vacation and/or sick leave during pandemics or other crises.

Rural health care organizations in resource-scarce areas often struggle to stay open, as funding may barely cover operating costs. These same facilities may have exceeded capacity during the pandemic due to lower local rates of COVID-19 vaccinations. To maintain quality of care, it is critical to implement cost-effective HCW wellness strategies and identify HCWs that may be at the highest risk of psychological distress. The results of the present study suggest that provision of services to treat depression and anxiety may reduce CF. Given the low usage of wellness resources during the pandemic that our findings revealed, future research is needed to identify barriers to the use of wellness resources and mechanisms to improve access to mental health services. Even has the pandemic is winding down, health care systems continue to experience acute staffing shortages and hence our results may be relevant to transforming work cultures by elevating the importance of wellness and prioritizing HCW preferences, such as requests for time off to use wellness resources.

### Study limitations

This study had the following limitations: 1) We did not measure HCWs’ knowledge about or attitudes towards receiving mental health treatment. 2) The sample included HCWs from both Internal Medicine and Psychiatry, but results might differ between these groups. 3) The survey did not differentiate between HCWs working on the front line in COVID units and those working elsewhere. 4) This was a cross-sectional survey with data collected at only one time point and early in the pandemic. Therefore, they do not reflect how wellness needs of HCWs may have changed throughout the course of the pandemic. 5) We surveyed HCWs from only one health service, limiting generalizability.

## Conclusion

Our study found low levels of CF with high CS in rural HCWs during the COVID 19 pandemic. Factors associated with low levels of CF included resilience and social support. Evidenced-based strategies that foster resilience and continue to improve social support may reduce the risk of psychological distress in rural HCWs. Rural health care organizations should build a culture of wellness, identifying strategies that ensure their employees know they are valued and supported. Importantly, efforts should focus on changing organizational culture by encouraging and promoting existing programs and the development of new resources. Deployment of needs-assessment surveys, like the one used in this study, will allow health care organizations to tailor efforts to improve HCW wellness to their employees’ needs and may ultimately lead to reduced CF, improved CS, and prevention of attrition within the healthcare professions.
